# 2-(Pyridin-4-yl)-1*H*-benzimidazole

**DOI:** 10.1107/S160053681301252X

**Published:** 2013-05-11

**Authors:** David K. Geiger, Christopher J. Bond

**Affiliations:** a Department of Chemistry, State University of New York-College at Geneseo, 1 College Circle, Geneseo, NY 14454, USA

## Abstract

The title compound, C_12_H_9_N_3_, is an unhydrated analogue of the previously reported trihydrate. The mol­ecule is essentially planar, with a 3.62 (11)° angle between the pyridine and benzimidazole planes. In the crystal, N—H⋯N hydrogen bonds result in chains of mol­ecules parallel to [010], which are additionally linked by weak π–π stacking inter­actions [centroid–centroid distance = 3.7469 (17) Å], resulting in extended sheets of molecules parallel to (103).

## Related literature

For the structure of the trihydrate of the title compound, see: Huang *et al.* (2004 ▶)
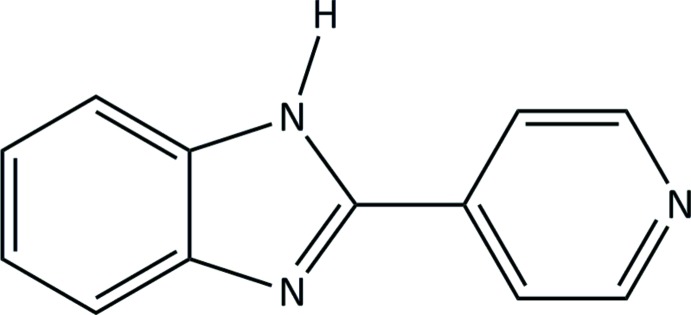


## Experimental

### 

#### Crystal data

C_12_H_9_N_3_
*M* *_r_* = 195.22
Monoclinic, 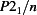

*a* = 6.0602 (14) Å
*b* = 11.610 (3) Å
*c* = 13.892 (4) Å
β = 101.838 (8)°
*V* = 956.6 (4) Å^3^
*Z* = 4
Mo *K*α radiation
μ = 0.09 mm^−1^
*T* = 200 K
0.50 × 0.20 × 0.20 mm

#### Data collection

Bruker SMART X2S CCD diffractometer
Absorption correction: multi-scan (*SADABS*; Bruker, 2010 ▶) *T*_min_ = 0.53, *T*_max_ = 0.98
4809 measured reflections
1699 independent reflections
1125 reflections with *I* > 2σ(*I*)
*R*_int_ = 0.058

#### Refinement

*R*[*F* ^2^ > 2σ(*F* ^2^)] = 0.046
*wR*(*F* ^2^) = 0.128
*S* = 0.96
1699 reflections
140 parameters
H atoms treated by a mixture of independent and constrained refinement
Δρ_max_ = 0.26 e Å^−3^
Δρ_min_ = −0.25 e Å^−3^

### 

Data collection: *APEX2* (Bruker, 2010 ▶); cell refinement: *SAINT* (Bruker, 2010 ▶); data reduction: *SAINT*; program(s) used to solve structure: *SHELXS97* (Sheldrick, 2008 ▶); program(s) used to refine structure: *SHELXL97* (Sheldrick, 2008 ▶); molecular graphics: *PLATON* (Spek, 2009 ▶) and *Mercury* (Macrae *et al.*, 2008 ▶); software used to prepare material for publication: *publCIF* (Westrip, 2010 ▶).

## Supplementary Material

Crystal structure: contains datablock(s) global, I. DOI: 10.1107/S160053681301252X/im2430sup1.cif

Structure factors: contains datablock(s) I. DOI: 10.1107/S160053681301252X/im2430Isup2.hkl

Supplementary material file. DOI: 10.1107/S160053681301252X/im2430Isup3.mol

Supplementary material file. DOI: 10.1107/S160053681301252X/im2430Isup4.cml

Additional supplementary materials:  crystallographic information; 3D view; checkCIF report

## Figures and Tables

**Table 1:** Hydrogen-bond geometry (Å, °)

*D*—H⋯*A*	*D*—H	H⋯*A*	*D*⋯*A*	*D*—H⋯*A*
N1—H1⋯N3^i^	0.99 (2)	1.96 (2)	2.924 (2)	165 (2)

Symmetry code: (i) 
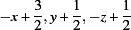
.

**Fig. 1.**
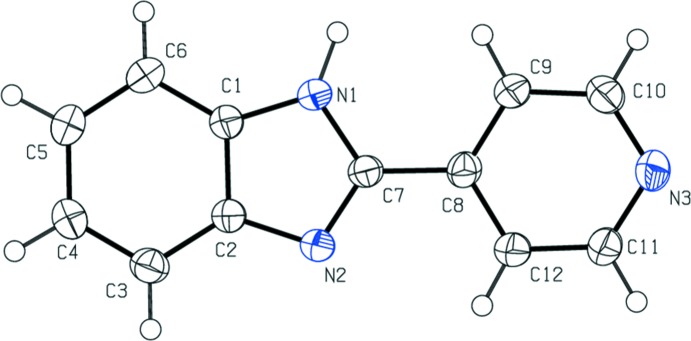
Perspective view of the title compound. Thermal parameters are drawn at the 50% probability level.

**Fig. 2.**
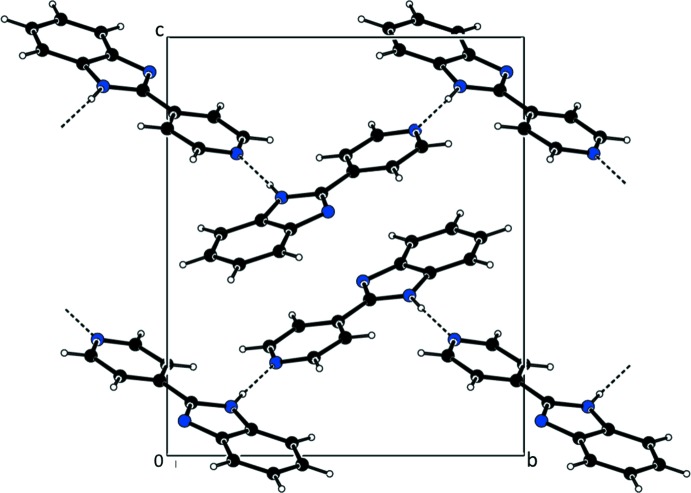
Packing diagram down [1 0 0] displaying the H-bonding network.

## supplementary crystallographic information

### Comment

Single crystals of the title compound were obtained by slow evaporation of an ethylacetate solution.

Figure 1 shows a perspective view of the title compound with the atom numbering scheme. The non-hydrogen atoms of the molecule are essentially planar with a r. m. s. deviation of 0.0308 Å. The maximum deviation is 0.0480 (18) Å for C12. The pyridine and benzimidazole planes exhibit a dihedral angle of 3.62 (11)°, which is similar to the value (2.8 (1)°) reported for the trihydrate analogue (Huang *et al.*, 2004).

Hydrogen-bonding interactions involving the benzimidazole N—H and the pyridine result in chains of molecules parallel to [0 1 0]. Figure 2 shows a packing diagram with the hydrogen-bonded chains displayed. The trihydrate (Huang *et al.*, 2004) hydrogen-bonding network is much more extensive, involving the three waters of hydration, the benzimidazole amine group and the pyridine. As seen in figure 2, pairs of molecules related by an inversion center exhibit π stacking. The spacing between the mean planes formed by the molecules is 3.43 Å. The shortest internuclear separation between related molecules is 3.460 (2) Å (N2···C7).

### Experimental

The title compound was prepared by stirring 0.373 g (3.45 mmole) *o*-phenylenediamine, 0.67 ml (7.1 mmole) 4-pyridinecarboxaldehyde, and 0.75 g NH_4_Cl in 25 ml CHCl_3_ for 5 days at room temperature. After removal of the solvent, the crude product mixture was extracted with water and ethylacetate and the solvent was removed from the organic phase. The resulting solid was passed through a short silica column using a 30:70 mixture of hexanes: ethyl acetate, yielding 0.442 g of solid that contained two components based on TLC analysis was obtained. The mixture was passed through a second silica column using 90:10 ethyl acetate: ethanol. The first component isolated was the title compound (0.380 g, 1.95 mmole, 56% yield). ^1^H NMR (400 MHz, *DMSO-d_6_*): δ, 7.27 (m, 2H), 7.64 (m, 2H), 8.09 (d, 2H), 8.75, (d, 2H). ^13^C NMR (*DMSO-d_6_*): δ, 120.89, 123.52, 137.77, 149.06, 150.67, 163.55.

### Refinement

All hydrogen atoms were observed in difference Fourier maps. The H atoms bonded to carbon were refined using a riding model with C—H = 0.95 Å and *U*_iso_ = 1.2*U*_eq_(C). The coordinates and isotropic thermal parameters of the amine H atom were refined without constraints.

### Crystal data

**Table:**

C_12_H_9_N_3_	*F*(000) = 408
*M**_r_* = 195.22	*D*_x_ = 1.356 Mg m^−^^3^
Monoclinic, *P*2_1_/*n*	Mo *K*α radiation, λ = 0.71073 Å
*a* = 6.0602 (14) Å	Cell parameters from 1098 reflections
*b* = 11.610 (3) Å	θ = 2.3–24.2°
*c* = 13.892 (4) Å	µ = 0.09 mm^−^^1^
β = 101.838 (8)°	*T* = 200 K
*V* = 956.6 (4) Å^3^	Prism, clear yellow
*Z* = 4	0.50 × 0.20 × 0.20 mm

### Data collection

**Table:**

Bruker SMART X2S CCD diffractometer	1699 independent reflections
Radiation source: XOS X-beam microfocus source	1125 reflections with *I* > 2σ(*I*)
Doubly curved silicon crystal monochromator	*R*_int_ = 0.058
Detector resolution: 8.3330 pixels mm^-1^	θ_max_ = 25.4°, θ_min_ = 3.0°
ω scans	*h* = −7→7
Absorption correction: multi-scan (*SADABS*; Bruker, 2010)	*k* = −13→8
*T*_min_ = 0.53, *T*_max_ = 0.98	*l* = −16→16
4809 measured reflections	

### Refinement

**Table:**

Refinement on *F*^2^	Primary atom site location: structure-invariant direct methods
Least-squares matrix: full	Secondary atom site location: difference Fourier map
*R*[*F*^2^ > 2σ(*F*^2^)] = 0.046	Hydrogen site location: inferred from neighbouring sites
*wR*(*F*^2^) = 0.128	H atoms treated by a mixture of independent and constrained refinement
*S* = 0.96	*w* = 1/[σ^2^(*F*_o_^2^) + (0.0664*P*)^2^] where *P* = (*F*_o_^2^ + 2*F*_c_^2^)/3
1699 reflections	(Δ/σ)_max_ < 0.001
140 parameters	Δρ_max_ = 0.26 e Å^−^^3^
0 restraints	Δρ_min_ = −0.25 e Å^−^^3^

### Special details

**Table:**

Experimental. The H atoms bonded to carbon were refined using a riding model with C—H = 0.95 Å and *U*_iso_ = 1.2*U*_eq_(C). The coordinates and isotropic thermal parameters of the amine H atom were refined freely.
Geometry. All e.s.d.'s (except the e.s.d. in the dihedral angle between two l.s. planes) are estimated using the full covariance matrix. The cell e.s.d.'s are taken into account individually in the estimation of e.s.d.'s in distances, angles and torsion angles; correlations between e.s.d.'s in cell parameters are only used when they are defined by crystal symmetry. An approximate (isotropic) treatment of cell e.s.d.'s is used for estimating e.s.d.'s involving l.s. planes.
Refinement. Refinement of *F*^2^ against ALL reflections. The weighted *R*-factor *wR* and goodness of fit *S* are based on *F*^2^, conventional *R*-factors *R* are based on *F*, with *F* set to zero for negative *F*^2^. The threshold expression of *F*^2^ > σ(*F*^2^) is used only for calculating *R*-factors(gt) *etc*. and is not relevant to the choice of reflections for refinement. *R*-factors based on *F*^2^ are statistically about twice as large as those based on *F*, and *R*- factors based on ALL data will be even larger.

### Fractional atomic coordinates and isotropic or equivalent isotropic displacement parameters (Å^2^)

**Table:**

	*x*	*y*	*z*	*U*_iso_*/*U*_eq_	
N1	0.4979 (3)	0.67895 (13)	0.38292 (14)	0.0331 (5)	
H1	0.618 (4)	0.7118 (19)	0.3528 (19)	0.062 (7)*	
N2	0.2493 (3)	0.54899 (13)	0.41681 (13)	0.0303 (5)	
N3	0.7075 (3)	0.30522 (13)	0.22238 (14)	0.0357 (5)	
C1	0.3669 (3)	0.73593 (15)	0.43732 (16)	0.0294 (5)	
C2	0.2130 (3)	0.65460 (15)	0.45835 (16)	0.0286 (5)	
C3	0.0506 (3)	0.68515 (17)	0.51158 (17)	0.0338 (6)	
H3	−0.0548	0.6305	0.5259	0.041*	
C4	0.0490 (3)	0.79775 (16)	0.54271 (18)	0.0389 (6)	
H4	−0.0594	0.8211	0.5794	0.047*	
C5	0.2039 (3)	0.87851 (17)	0.52133 (19)	0.0412 (6)	
H5	0.1968	0.9556	0.5434	0.049*	
C6	0.3650 (4)	0.84967 (16)	0.46965 (18)	0.0398 (6)	
H6	0.471	0.9046	0.4563	0.048*	
C7	0.4191 (3)	0.56767 (15)	0.37324 (16)	0.0284 (5)	
C8	0.5194 (3)	0.47905 (15)	0.32041 (16)	0.0295 (5)	
C9	0.7077 (4)	0.49937 (16)	0.28180 (18)	0.0368 (6)	
H9	0.7769	0.5732	0.2878	0.044*	
C10	0.7947 (4)	0.41118 (17)	0.23429 (18)	0.0395 (6)	
H10	0.9251	0.4268	0.2084	0.047*	
C11	0.5260 (3)	0.28667 (16)	0.26116 (17)	0.0352 (6)	
H11	0.4609	0.2119	0.2545	0.042*	
C12	0.4278 (3)	0.36867 (15)	0.30996 (17)	0.0336 (6)	
H12	0.2992	0.3503	0.3362	0.04*	

### Atomic displacement parameters (Å^2^)

**Table:**

	*U*^11^	*U*^22^	*U*^33^	*U*^12^	*U*^13^	*U*^23^
N1	0.0314 (10)	0.0239 (9)	0.0474 (13)	−0.0038 (7)	0.0161 (9)	−0.0026 (8)
N2	0.0291 (10)	0.0267 (9)	0.0376 (12)	−0.0014 (7)	0.0130 (9)	0.0002 (7)
N3	0.0372 (11)	0.0305 (9)	0.0406 (13)	0.0062 (8)	0.0110 (9)	−0.0012 (8)
C1	0.0284 (11)	0.0270 (10)	0.0342 (14)	0.0005 (8)	0.0098 (10)	−0.0002 (9)
C2	0.0270 (11)	0.0259 (10)	0.0334 (13)	−0.0006 (8)	0.0075 (10)	0.0002 (9)
C3	0.0296 (12)	0.0350 (11)	0.0381 (15)	−0.0014 (9)	0.0104 (11)	0.0019 (9)
C4	0.0375 (13)	0.0379 (12)	0.0454 (16)	0.0028 (10)	0.0182 (12)	−0.0031 (10)
C5	0.0478 (14)	0.0292 (11)	0.0501 (16)	−0.0011 (9)	0.0185 (12)	−0.0083 (10)
C6	0.0426 (14)	0.0274 (11)	0.0519 (17)	−0.0073 (9)	0.0158 (12)	−0.0059 (10)
C7	0.0261 (11)	0.0242 (10)	0.0353 (14)	−0.0011 (8)	0.0074 (10)	0.0010 (9)
C8	0.0279 (12)	0.0241 (9)	0.0362 (14)	0.0036 (8)	0.0056 (10)	0.0019 (9)
C9	0.0380 (13)	0.0252 (10)	0.0511 (17)	−0.0010 (9)	0.0183 (12)	0.0008 (9)
C10	0.0374 (13)	0.0362 (11)	0.0496 (16)	0.0028 (9)	0.0201 (12)	0.0032 (10)
C11	0.0339 (13)	0.0276 (10)	0.0450 (16)	0.0005 (9)	0.0105 (11)	−0.0047 (9)
C12	0.0297 (12)	0.0293 (11)	0.0437 (15)	−0.0021 (9)	0.0119 (11)	−0.0013 (9)

### Geometric parameters (Å, º)

**Table:**

N1—C1	1.373 (3)	C4—H4	0.95
N1—C7	1.374 (2)	C5—C6	1.367 (3)
N1—H1	0.99 (2)	C5—H5	0.95
N2—C7	1.315 (3)	C6—H6	0.95
N2—C2	1.392 (2)	C7—C8	1.466 (3)
N3—C10	1.336 (2)	C8—C9	1.377 (3)
N3—C11	1.338 (3)	C8—C12	1.392 (3)
C1—C6	1.396 (3)	C9—C10	1.380 (3)
C1—C2	1.399 (3)	C9—H9	0.95
C2—C3	1.393 (3)	C10—H10	0.95
C3—C4	1.378 (3)	C11—C12	1.373 (3)
C3—H3	0.95	C11—H11	0.95
C4—C5	1.401 (3)	C12—H12	0.95
			
C1—N1—C7	106.18 (16)	C5—C6—H6	121.5
C1—N1—H1	127.4 (13)	C1—C6—H6	121.5
C7—N1—H1	126.4 (14)	N2—C7—N1	113.51 (17)
C7—N2—C2	104.53 (15)	N2—C7—C8	123.97 (16)
C10—N3—C11	115.83 (18)	N1—C7—C8	122.52 (18)
N1—C1—C6	132.60 (19)	C9—C8—C12	117.48 (19)
N1—C1—C2	105.96 (16)	C9—C8—C7	122.43 (17)
C6—C1—C2	121.44 (19)	C12—C8—C7	120.06 (19)
N2—C2—C3	129.31 (18)	C8—C9—C10	119.18 (18)
N2—C2—C1	109.83 (18)	C8—C9—H9	120.4
C3—C2—C1	120.84 (18)	C10—C9—H9	120.4
C4—C3—C2	117.37 (19)	N3—C10—C9	124.2 (2)
C4—C3—H3	121.3	N3—C10—H10	117.9
C2—C3—H3	121.3	C9—C10—H10	117.9
C3—C4—C5	121.4 (2)	N3—C11—C12	124.24 (18)
C3—C4—H4	119.3	N3—C11—H11	117.9
C5—C4—H4	119.3	C12—C11—H11	117.9
C6—C5—C4	121.96 (19)	C11—C12—C8	119.1 (2)
C6—C5—H5	119.0	C11—C12—H12	120.5
C4—C5—H5	119.0	C8—C12—H12	120.5
C5—C6—C1	117.04 (19)		

### Hydrogen-bond geometry (Å, º)

**Table:**

*D*—H···*A*	*D*—H	H···*A*	*D*···*A*	*D*—H···*A*
N1—H1···N3^i^	0.99 (2)	1.96 (2)	2.924 (2)	165 (2)

Symmetry code: (i) −*x*+3/2, *y*+1/2, −*z*+1/2.

## References

[bb1] Bruker (2010). *APEX2*, *SAINT* and *SADABS* Bruker AXS Inc., Madison, Wisconsin, USA.

[bb2] Huang, X.-C., Zeng, M.-H. & Ng, S. W. (2004). *Acta Cryst.* E**60**, o939–o940.

[bb3] Macrae, C. F., Bruno, I. J., Chisholm, J. A., Edgington, P. R., McCabe, P., Pidcock, E., Rodriguez-Monge, L., Taylor, R., van de Streek, J. & Wood, P. A. (2008). *J. Appl. Cryst.* **41**, 466–470.

[bb4] Sheldrick, G. M. (2008). *Acta Cryst.* A**64**, 112–122. 10.1107/S0108767307043930 18156677

[bb5] Spek, A. L. (2009). *Acta Cryst.* D**65**, 148–155. 10.1107/S090744490804362X PMC2631630 19171970

[bb6] Westrip, S. P. (2010). *J. Appl. Cryst.* **43**, 920–925.

